# Boerhaave Syndrome—Narrative Review

**DOI:** 10.3390/diagnostics15192463

**Published:** 2025-09-26

**Authors:** Dragos Predescu, Florin Achim, Bogdan Socea, Alexandru Rotariu, Alex-Claudiu Moraru, Anthony Rasuceanu, Carmen Constantin, Cristian Gelu Rosianu, Adrian Constantin

**Affiliations:** 1 Faculty of Medicine, Carol Davila University of Medicine and Pharmacy Bucharest, 050474 Bucharest, Romania; drpredescu@yahoo.com (D.P.); bogdan.socea@umfcd.ro (B.S.); alexandru.rotariu95@yahoo.com (A.R.); alex.claudiu.moraru@gmail.com (A.-C.M.); rasuceanu.anthony@gmail.com (A.R.); cioceacarmen@yahoo.com (C.C.); rosianu_cristian@yahoo.com (C.G.R.); dradiconstantin@yahoo.com (A.C.); 2 Department of Esophageal and General Surgery, Sf. Maria Clinical Hospital, 011192 Bucharest, Romania; 3 Department of Surgery, Sf. Pantelimon Clinical Emergency Hospital, 021659 Bucharest, Romania; 4 Department of Anesthesia and Intensive Care, Sf. Maria Clinical Hospital, 011192 Bucharest, Romania; 5 Department of Gastroenterology, Sf. Maria Clinical Hospital, 011192 Bucharest, Romania

**Keywords:** Boerhaave syndrome, esophageal perforation, esophageal rupture, mediastinitis, endoscopic treatment

## Abstract

Boerhaave syndrome (BS) and subsequent septic mediastinitis represent a complex cascade of events from esophageal perforation to septic shock. The pathophysiology involves chemical injury, polymicrobial contamination, cytokine storm, endothelial dysfunction, coagulation disorders, and ultimately multiple organ failure. Understanding these mechanisms is crucial for proper therapeutic management that can interrupt this lethal sequence. Due to the complexity of this condition, it is almost impossible to develop a feasible treatment protocol for every situation. The article, through a literature review, evaluates the pathophysiological mechanisms, the consequences of spontaneous esophageal rupture, as well as the therapeutic techniques available for these situations. These elements are the basis of the management of spontaneous esophageal rupture, which involves adapting and customizing the treatment for each patient.

## 1. Introduction

Esophageal perforations represent an extreme pathology requiring sustained medical effort, significant costs, and a multidisciplinary approach for both diagnosis and treatment. Etiologically, they may be iatrogenic, traumatic, caused by foreign bodies, related to other esophageal diseases, or spontaneous [1]. Iatrogenic perforations are the most frequent, accounting for approximately 52% of cases. BS is responsible for 8–56% of esophageal perforations [2]. BS is a rare condition, characterized by rupture of the esophageal wall following a sudden increase in intraesophageal pressure, most often secondary to vomiting efforts.

**Figure 1:**
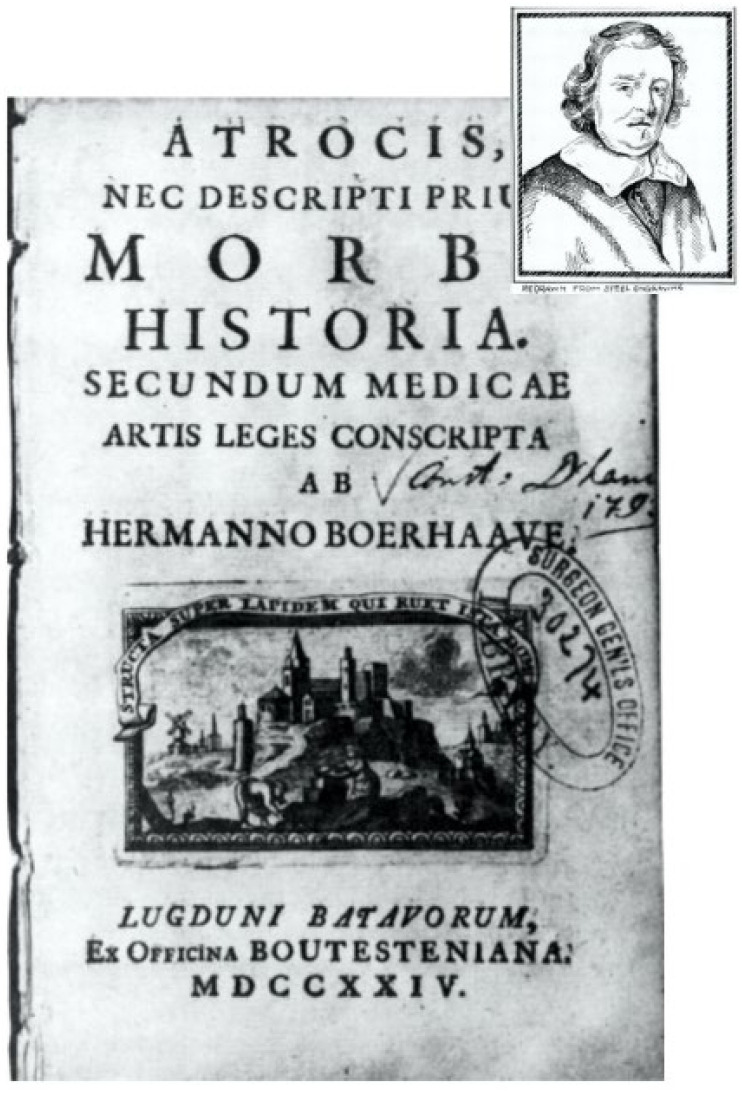
“An Account of the Life and Writings of Herman Boerhaave”—monograph. The smaller image shows Hermann Boerhaave, Doctor of Philosophy and Medicine, Professor of the Theory and Practice of Physic, and of Botany and Chemistry in the University of Leyden; President of the Surgical College in Leyden, Fellow of the Royal Society in London, and Fellow of the Royal Academy in Paris.

Historically, the syndrome was described by Hermann Boerhaave in 1724, with the first reported case being Baron Jan von Wassenaer, a Danish admiral (Figure 1). He was known for his excessive indulgence in food and drink, and after a heavy meal associated with significant alcohol intake, he experienced a violent episode of vomiting, followed by severe left-sided chest pain, rapid deterioration of his general condition, coma, and ultimately, death [3]. The pathophysiology of esophageal rupture originates from a paroxysmal rise in esophageal pressure in a “closed-tube” situation, typically following intense, violent efforts such as vomiting. However, it has also been reported in other circumstances, such as during defecation, childbirth, weightlifting, etc. [4]. A certain profile can often be identified: predominantly male patients, frequently obese, gourmands and alcohol consumers (particularly beverages of fermentation, such as beer, cider, and wine).

In these conditions, spontaneous rupture of the esophagus often follows heavy meals combined with substantial alcohol consumption, leading to violent vomiting episodes [5].

Particular cases of BS have been described in patients with diabetic ketoacidosis, where vomiting episodes also precipitate esophageal rupture. These cases are especially challenging due to the complex underlying physiopathology. Additionally, spontaneous esophageal perforations have been reported during renal colic, when the digestive symptoms include violent vomiting [6].

Diagnosis is challenging but crucial. If made within the first 24 h, mortality can be reduced to 6.2%. The consequences of spontaneous esophageal rupture are severe, and the need for fast diagnosis and initiation of effective treatment is an uncontested consensus in the medical community. Between 10 and 25% of cases are treated within the first 24 h, while 40–60% are treated after 48 h [7].

## 2. Objectives

The small number of cases reported in the literature makes it difficult to develop a rigorous, reproducible treatment protocol for BS, particularly since most publications consist only of case reports. The evaluation of pathophysiological consequences due to leakage of digestive contents into the mediastinum secondary to spontaneous esophageal rupture, along with the review of current diagnostic and therapeutic possibilities in such situations, are fundamental to the management of BS. Prompt decision-making is absolutely essential, and adaptation to each individual case is driven by the complexity of this rare pathological entity, which is addressed almost exclusively in centers of excellence specializing in esophageal pathology.

This paper examines current hypotheses regarding the etio-physiopathogenesis of BS, the impact of sepsis of mediastinal origin and diagnosis strategies associated with therapeutic challenges. Perspectives on diagnostic, endoscopic intervention and surgical management from a high-volume tertiary center are discussed to guide future research and clinical practice.

Sf. Maria Clinical Hospital is a tertiary center specialized in esophageal pathology. Over a period of 20 years, between 1 January 2005 and 31 August 2024, a total of 6 cases of BS were diagnosed and treated in our department, of which 1 case was pathological esophagus (eosinophilic esophagitis). All cases required interventions under general anesthesia in the operating room.

- In two cases, the resolution was strictly gastroenterological (stenting) combined with sustained intensive care support.
- In other two cases, surgical esophagectomy was necessary, followed by esophageal reconstruction.
- In the fifth case, a combined approach was used—endoscopical (stenting) and surgical (thoraco-laparoscopic) interventions performed in the same therapeutic session, a so-called “rendez-vous” procedure.
- In the sixth case, the patient was known to have eosinophilic esophagitis, and stenting, PEG placement and antibiotic therapy were given.

All six patients survived following treatment.

## 3. Search Strategy

**Figure 2:**
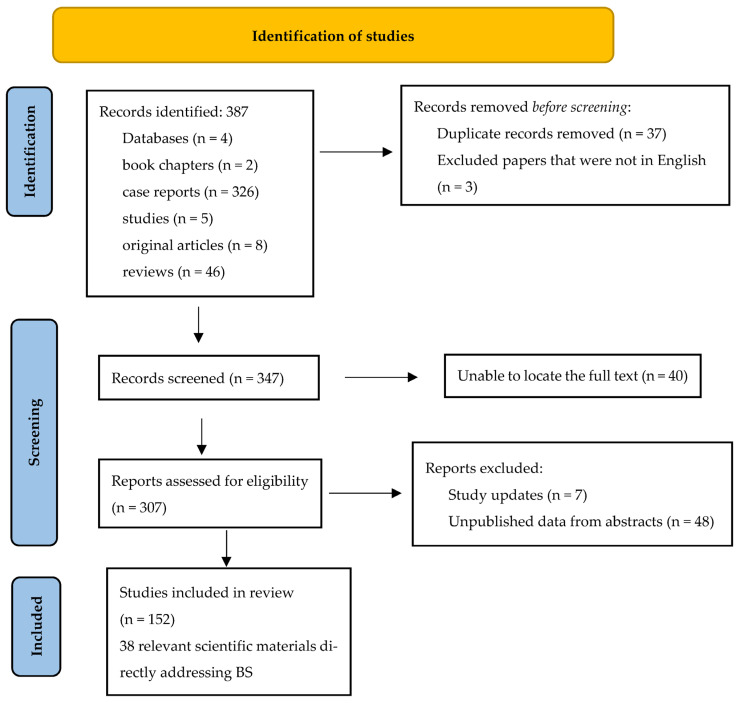
The PRISMA diagram.

The search strategy was based on keywords and phrases used in search engines PubMed Central (PMC), Cochrane Library, Embase (Excerpta Medica Database) and MEDLINE Complete (EBSCO) in the last 25 years, specifically the following terms: “esophageal perforation” and “Boerhaave syndrome”. Following the search, 387 scientific materials related to the topic were identified, including (Figure 2): book chapters (2), case reports (326), original articles (8), studies (5), and reviews (46). To refine the selection specifically toward the theme of Boerhaave syndrome–esophageal perforation, the “Advanced option” was used, introducing additional criteria such as: “mediastinal sepsis,” “BS diagnosis,” “diagnostic and therapeutic endoscopy,” “surgical treatment,” and “systemic therapy”. This allowed for the use of a “search history” feature and facilitated the combination of individual searches using Boolean operators “AND” and “OR.” Parentheses were automatically placed around each set of terms to preserve the logical structure of the search. As a result, 38 scientific materials directly addressing BS were identified. Additional articles from the initial reference list, considered relevant to the topic, were also included. Two authors (A.C. and D.P.) independently selected the articles deemed relevant, with a preference for high-ranking journals and articles written in English. The decision to select an article was made by agreement between the two reviewers. The number of citations of each article was also an important selection criterion. Each article was allocated a different review time based on its type: 15 min for clinical cases, and 30–60 min for reviews, original articles, and book chapters.

Differences between reviewers were discussed, and if a consensus could not be reached, a third reviewer (F.A.) was consulted for recommendation. Unpublished data, abstracts from conference proceedings, and non-English-language works were excluded from the final selection.

## 4. Incidence

BS is considered a rare condition, with an incidence of 3.1 cases per 1,000,000 per year, primarily described in middle-aged men with a history of excessive food intake and alcohol consumption [8]. Although the majority of esophageal perforations are iatrogenic, BS accounts for approximately 15% of all esophageal perforations, specifically including cases without pre-existing esophageal lesions [9].

There is also a subgroup of spontaneous esophageal perforations occurring in patients with documented pre-existing esophageal pathology, such as eosinophilic esophagitis, drug-induced esophagitis, Barrett’s esophagus, or infectious esophageal ulcers. The incidence is significantly higher in men, with a male-to-female ratio ranging from 2:1 to 5:1, and there are no racial differences reported [10].

Although the highest incidence is documented in men during their 6th and 7th decades of life, cases have been recorded both in newborns and in patients over 90 years of age. Children aged 1 to 17 years are the least affected demographic group [11].

## 5. Pathophysiology of Esophageal Rupture

BS is essentially the consequence of a lack of coordination between the upper and lower esophageal sphincters, resulting in transmural tearing of the distal esophageal wall due to increased intragastric pressure transmitted to the esophagus during vomiting episodes [12]. In very rare cases, the perforation may occur at the cervical level [13].

Esophageal rupture is followed by contamination of the mediastinum and pleural cavity with gastroesophageal contents (digestive secretions and food debris). Thoracic movement during respiration, with the resulting pressure fluctuations, disperses these effusions, exacerbating pleural and mediastinal contamination and contributing to the severity of the condition [14]. In the absence of medical and surgical intervention, bacterial infection and mediastinal necrosis will eventually lead to sepsis and multiple organ dysfunction syndrome (MODS) [6]. Reported mortality rates vary widely in the literature, ranging from 30% to 90% depending on the source [7,9].

Although the exact cause remains unknown, there is consensus that esophageal rupture results when a very rapid increase in intraesophageal pressure combines with proximal esophageal obstruction, a phenomenon typically encountered during vomiting episodes [15]. Experimental data obtained from fresh cadaver models, with esophageal clamping at the mid-esophagus, have shown that a pressure of 150 mmHg is sufficient to produce a rupture at the gastroesophageal junction [16].

In patients with spontaneous esophageal rupture, it is currently estimated that pressures up to 200 mmHg—or even higher—are reached, similar to nutcracker esophagus [17].

Within the context of BS, in approximately 90% of cases, the rupture occurs at the left wall of the distal esophagus. This predisposition is explained by anatomical and histological factors, namely:

- Thinning of the esophageal muscular layer at this level
- Weak points where neurovascular structures penetrate the distal esophageal wall, creating vulnerable zones prone to intraluminal hyperpressure
- Lack of support from neighboring structures
- The anterior curvature of the esophagus at the level of the left diaphragmatic crus [18].

Moreover, in-depth studies suggest that, concomitant with the barotrauma injury, there is a cranial traction phenomenon affecting the left edge of the distal esophagus, alongside a prolapse of the gastric mucosa into the esophageal lumen during vomiting episodes [15]. Furthermore, numerous reports describe aberrant motor phenomena of the digestive tract during vomiting episodes [19,20,21,22,23].

Endoscopic studies have demonstrated repeated prolapse of the gastric mucosa into the esophageal lumen during vomiting episodes in certain patients [24]. This phenomenon has been described in 1.8% of cases, most often involving the left margin of the esophagus, a topography that coincides with the most common site of esophageal ruptures seen in BS [15]. It remains difficult to determine whether this is just a coincidence or a real physiopathological component of the syndrome.

The average length of the laceration varies among different studies, generally between 2.2 and 3.3 cm. Similarly, depending on the source, reported esophageal wound lengths can vary widely, most often ranging between 1 and 8 cm, although there are cases where the esophageal rupture presents as a longitudinal tear involving almost the entire thoracic esophagus [25]. Regardless of its length, the mucosal tear is usually longer than the muscular layer rupture, a fact with direct implications for the choice and technique of surgical repair [17].

A case of spontaneous double perforation has been described, although it is difficult to confirm whether one of the perforations was truly spontaneous and not iatrogenic, discovered during a later evaluation [26,27]. Recurrent spontaneous rupture is very rare, with only a few cases reported [28,29,30,31].

There is a documented case in the literature of a triple recurrence, with each episode managed using a different therapeutic method [32]. Another highly unusual case reports two BS events in the same patient—one rupture in the intra-abdominal esophagus and another in the thoracic esophagus—considered unique in the literature [33].

On the other hand, it is crucial to differentiate BS from Mallory-Weiss syndrome, as they can occur under similar circumstances and initially present with comparable clinical signs.

However, the anatomical site and especially the depth of the lesion determine major differences in therapeutic management and prognosis:

- In BS, the lesion typically affects the left margin of the distal esophagus (in 90% of cases) and involves the full thickness of the esophageal wall;
- In contrast, in Mallory-Weiss syndrome, the laceration occurs at the level of the cardia and does not extend beyond the submucosa [4].

There is an ongoing discussion in the literature regarding the relationship between the two syndromes, with some authors suggesting that BS may represent an extension of Mallory-Weiss syndrome [34,35,36,37,38,39,40]. However, this relationship is difficult to support, primarily due to the different topographies involved (left wall of the distal esophagus vs. the cardia) and the near absence of properly documented intermediate cases. A review of the possible “transitional cases” identified only one documented case that could qualify for such scenario. All other cases considered “transitional” in the literature actually showed not only multiple extended lacerations into the submucosa of the cardia but also a simultaneous full-thickness laceration at the distal esophagus. Thus, the conclusion would be that the two syndromes are distinct pathological entities, and many of the so-called transitional cases are in fact situations where both syndromes occur simultaneously in the same patient [4].

This overlap would be explained by their shared etiopathogenic mechanism—namely, increased intraluminal pressure secondary to vomiting—although with some notable differences:

- In Mallory-Weiss syndrome, the damage results from repetitive vomiting episodes affecting primarily the cardiac portion of the stomach, including the esophagogastric junction;
- In BS, the rupture is full-thickness and occurs at the weakest point of the esophageal wall, typically beginning with a muscular layer tear, preceding mucosal rupture [41];

Clinically, there are also a series of differences between the two entities:

- Mallory-Weiss syndrome is characterized by mild pain and massive hematemesis, whereas BS presents with severe pain and mild hematemesis [7]

Despite these distinctions, opinions are not unanimous. The literature describes the case of a 45-year-old man who died due to a spontaneous esophageal rupture. Autopsy findings, along with anamnesis and histopathology (revealing acute and chronic inflammatory changes within the esophageal wall), suggested that the fatal esophageal perforation did not occur suddenly, but developed from a prior partial wall injury that had been present for several days—thus indicating a possible transition from an initial Mallory-Weiss lesion to a Boerhaave-type lesion [2]. Whether inflammation from the initial incomplete esophageal wall injury weakened the wall’s resistance, ultimately leading to complete rupture through continued vomiting episodes, remains a speculative hypothesis. Pressure variations in the mediastinum during breathing favor mediastinal contamination once the esophageal rupture has affected all layers.

The esophagus is separated from the mediastinal pleura by loose, poorly vascularized connective tissue, which offers little resistance to the ascending or descending spread of fluid and gas collections—upward toward the apex of the pleural dome and downward toward the diaphragm [41].

In the infra-azygo-aortic region, the thoracic esophagus occupies the entire space between the pericardium and the dorsal plane of the posterior mediastinum, an area known as Truesdale’s space, characterized by abundant, loosely arranged, poorly vascularized tissue, providing an excellent culture medium in the context of septic contamination [42].

In the inter-azygo-aortic region, the periesophageal tissue atmosphere includes the interpleural Morozov ligament (also known as the aorto-esophageal ligament), a morphologically well-represented structure through which the following pass: vessels supplying the subphrenic portion of the thoracic esophagus, lymphatic and neural formations [43]. Functionally, the Morozov ligament exhibits great plasticity, changing its position depending on the phases of respiration. The movement and reorientation of the Morozov interpleural ligament during respiration are further facilitated by the Montiero periesophageal sheath, into which the esophagus is telescoped. This sheath operates bi-dimensionally, allowing easy displacement of the esophagus, both vertically (moving together with the diaphragm) and antero-posteriorly (driven by sternal movements) [44,45].

## 6. Mediastinitis and Sepsis Syndrome

The initial lesion in BS is caused by the leakage of acidic gastric contents into the mediastinum, leading to chemical mediastinitis [46]. The acidic environment damages mediastinal tissues, releasing damage-associated molecular patterns (DAMPs) such as HMGB1, ATP, and DNA, which activate pattern recognition receptors (PRRs) on immune cells [47]. At the same time, the perforation allows bacteria from the gastrointestinal tract to enter the mediastinum, resulting in polymicrobial contamination [48].

The normal esophageal flora includes aerobic bacteria, most commonly Viridans group streptococci, members of the Enterobacteriaceae family (such as *Klebsiella pneumoniae*, *Escherichia coli*), and Staphylococcus aureus. Less frequently, non-fermentative Gram-negative bacteria like *Pseudomonas aeruginosa* can be isolated, found in approximately 10% of cases. Among anaerobic bacteria, the *Bacteroides* group is prominent, especially *Bacteroides fragilis*, but species such as *Prevotella*, *Porphyromonas*, *Fusobacterium*, and *Peptostreptococcus* spp. are also documented. Although fungal infections were previously considered rare, recent studies report the presence of *Candida* species in 58% of cases, with *Candida albicans* and *Candida glabrata* being the most frequently isolated [49,50]. Once microbial colonization occurs, following the initial chemical aggression, a septic phase rapidly establishes itself, becoming the main factor of disease progression.

The inflammatory response is triggered fast in the presence of mediastinitis and is mediated by Toll-like receptors (TLRs) and NOD-like receptors (NLRs). These receptors, especially TLR4 and TLR2, play a crucial role in recognizing pathogen-associated molecular patterns (PAMPs): Lipopolysaccharides (LPS), a major component of Gram-negative bacterial walls, and Peptidoglycan, found in the walls of Gram-positive bacteria. Following bacterial multiplication within the mediastinal focus, bacterial exotoxins also play an important pathogenic role [51,52].

The progression from mediastinitis to sepsis occurs within a few hours after perforation, as chemical mediastinitis and polymicrobial contamination lead to an initial hyperinflammatory response [46]. Mediastinal infection rapidly progresses to sepsis and organ dysfunction, depending on the size of the esophageal rupture, the volume of contents leaked from the esophageal lumen, and pre-existing conditions and comorbidities [53,54].

The immune response triggered by mediastinal contamination secondary to esophageal rupture involves the recruitment of neutrophils and macrophages to the site of infection. These immune cells produce reactive oxygen species (ROS) and antimicrobial peptides to combat pathogens [47,54].

The cytokine storm in mediastinitis triggered by Boerhaave’s syndrome is characterized by the excessive release of pro-inflammatory cytokines, including TNF-α, IL-1β, and IL-6. Endothelial cells activated by TNF-α, IL-1β, and IL-6 also produce pro-inflammatory cytokines and chemokines, further amplifying the inflammatory response [55].

Subsequent endothelial dysfunction is associated with increased vascular permeability, leading to hypotension and tissue edema [56].

The coagulation disorders in septic mediastinitis caused by spontaneous esophageal rupture are secondary to dysfunction in tissue factor regulation, procoagulant endothelial changes, PAI-1 suppression, and impaired fibrinolysis [57].

Septic shock induced by mediastinitis in Boerhaave’s syndrome is characterized by an important decrease in systemic vascular resistance (SVR). This decrease is primarily due to the release of inflammatory mediators and vasodilatory substances [58].

Cytokine-mediated myocardial depression is a defining feature of septic shock following massive mediastinal contamination from spontaneous esophageal rupture [59,60].

The Compensatory Anti-Inflammatory Response Syndrome (CARS) is a physiological response intended to counteract the initial hyperinflammatory phase of sepsis in Boerhaave’s syndrome. IL-10 and transforming growth factor-beta (TGF-β) are key cytokines involved in this process [61,62].

Immune paralysis in Boerhaave’s syndrome is a state of immunosuppression that often follows the initial hyperinflammatory phase of sepsis. This condition is characterized by T-cell depletion, reduced phagocytic activity, and compromised cytokine production [63]. The mechanisms underlying immune paralysis involve the excessive upregulation of immune checkpoint molecules such as programmed death protein-1 (PD-1) and its ligand (PD-L1), which inhibit T-cell activation and proliferation. Moreover, the persistent release of anti-inflammatory cytokines such as IL-10 and TGF-β contributes to the suppression of immune responses, increasing the risk of secondary infections [64]. Immune paralysis, coupled with the disruption of physical barriers such as the skin and mucous membranes, creates a favorable environment for the colonization and invasion by opportunistic pathogens. Secondary infections further exacerbate the inflammatory response, leading to a vicious cycle of inflammation, immune suppression, and organ dysfunction [61,64]. To block these stages of sepsis progression in a timely manner, it is essential to diagnose esophageal perforation as early as possible and evaluate subsequent complications.

## 7. Diagnosis

The specific clinical picture is considered to be the so-called Mackler’s triad, which involves vomiting, chest pain, and cervical subcutaneous emphysema. However, the appearance is usually sequential: it begins with vomiting, followed by the onset of chest pain, and, subsequently, the appearance of cervical emphysema [65]. These elements are clearly secondary to the rupture of the esophageal wall, with digestive and gaseous leakage into the mediastinum, and the spread of this leakage along mediastinal anatomical structures both caudally and cranially, reaching the cervical region.

On the other hand, these clinical elements, either individually or together, are not specific to esophageal rupture, as they are also encountered in a wide range of pathological entities from the digestive, cardiovascular, ENT, among others. Thus, the differential diagnosis includes perforated gastric or duodenal ulcer, acute myocardial infarction, pericarditis, pneumothorax, pulmonary embolism, diaphragmatic hernia, dissecting aortic aneurysm, acute pancreatitis, and perforative ENT pathologies [66].

Moreover, although difficult, rapid diagnosis is essential, as mortality can reach up to 40%. Prompt diagnosis and appropriate treatment instituted within the first 24 h offer a 75% survival rate [6]. The prognosis deteriorates proportionally with the time elapsed from the onset of the esophageal rupture to the establishment of an effective treatment. A delay of 48 h reduces the survival rate to 50%, and after 72 h, survival drops to 10% [67].

Although in most cases the clinical presentation suggests the severity of the pathology—with the patient presenting as tachypneic, tachycardic, and potentially even in hemodynamic shock—unfortunately, the full triad of Mackler’s clinical signs is relatively rarely encountered, appearing in 20–50% of cases depending on the source [66,68].

An explanation for this low percentage may be found in the succession of pathophysiological events in Boerhaave’s syndrome. Vomiting dominates the initial clinical presentation. Vomiting causes the rupture of the esophageal wall, which in turn leads to contamination of the mediastinum with fluid leakage (digestive secretions and food content, clinically manifesting as pain of varying intensity. Although air leakage occurs simultaneously with fluid leakage, it requires a variable amount of time to manifest as subcutaneous emphysema at the cervical level.

Thus, it can be speculated that if diagnosis is established early, it could be before the full clinical triad is complete. Moreover, individual pain sensitivity thresholds differ, and recent significant alcohol consumption—frequently described in these patients—can elevate the pain threshold. Additionally, the common “pattern” in these patients makes it difficult to obtain proper information about the medical history. These are often uncooperative, intoxicated, and confused patients. Depending on the time elapsed since onset, they may be in shock, obtunded, or even comatose. In such cases, information provided by witnesses can be crucial.

In a recent Australian study [69], chest pain was present in 80% of cases, vomiting in 87%, and subcutaneous emphysema in 27%. In other reports, the causal relationship between vomiting and esophageal pathology is difficult to establish, since approximately 25–45% of patients do not report vomiting at hospital presentation [70].

Among the clinical triad, subcutaneous emphysema is considered the most pathognomonic element for esophageal rupture, but even this clinical marker does not exceed 60% of cases, according to the most optimistic statistics [71].

The heterogeneity of the clinical presentation, beyond Mackler’s triad, is thus a reality, and all clinical elements must be carefully identified and interpreted by a skilled medical team, as some signs may be less “loud” but still highly suggestive. The patient may present with decreased breath sounds on the side of the perforation or Hamman’s sign—a crunching sound synchronous with heartbeats [72,73]. Although described as exceptional cases, spontaneous perforations of the proximal esophagus following vomiting efforts can lead to cervical pain, dysphagia, and dysphonia [6].

Particular attention must be given to chest pain in Boerhaave’s syndrome. Although often severe and violent, it is non-specific in terms of topography and even intensity, and it carries the risk of misleading the medical team toward an incorrect diagnosis. On one hand, the intensity of chest pain can overshadow other clinical signs which, if correlated, could more accurately establish the diagnosis. On the other hand, the suspicion of acute coronary syndrome (ACS) triggered by the sudden onset of chest pain is understandable, given the high incidence of coronary pathologies, the somewhat similar patient profiles (middle-aged men, history of heavy eating, alcohol abuse, possibly smoking), and the relatively similar contexts triggering both conditions. Moreover, cases have been reported where Boerhaave’s syndrome presented with ST-segment changes on ECG [70]. Specifically, patients with spontaneous esophageal rupture have been admitted under suspicion of ST-elevation myocardial infarction (STEMI), although in none of these cases was there an actual increase in cardiac enzymes [74]. Additionally, other cases in the literature have described ST-segment elevations in patients with pneumomediastinum of various etiologies. Although the exact cause of these ECG changes is not fully understood, it is believed to be related to cardiac displacement due to mediastinal widening, right ventricular enlargement, and pericardial air collection exerting a mass effect on the heart [75].

These diagnostic errors have a particularly serious risk, especially because they may lead to delays in conducting urgent digestive tract investigations, which would be deemed unnecessary if acute cardiac pathology is incorrectly presumed. The immediate consequence is a delay in the institution of appropriate treatment for Boerhaave’s syndrome, resulting in a dramatically worsened prognosis.

Cardiac complications secondary to esophageal rupture are probably due to local inflammation following gastric content leakage into the mediastinum. Cases of third-degree atrioventricular block secondary to Boerhaave’s syndrome have also been described, although the underlying mechanism remains largely speculative [76].

## 8. Paraclinical Investigations

*Ultrasound* is an established tool for assessing hemodynamically unstable patients in emergency settings. In the diagnosis of pneumothorax or hydropneumothorax, ultrasound is known to have a sensitivity greater than 90% [73].

In Boerhaave’s syndrome, ultrasound may reveal mediastinal air collections, most characteristically in the shape of an A sign around the pericardium, or merely subcutaneous emphysema [77]. There are a limited number of reported cases where Boerhaave’s syndrome was diagnosed using ultrasound. One such report describes a case where ultrasound, performed on a vomiting patient, revealed a fluid collection with internal echoes compatible with pneumo-hemothorax, which was later proven to be secondary to spontaneous esophageal rupture [78]. Ultrasound is non-invasive, can be performed on poorly cooperative patients, and can be done with portable devices (bedside ultrasound) [73]. In the literature, a case has been described where pneumomediastinum was detected through bedside ultrasound, raising suspicion of Boerhaave’s syndrome, later confirmed [79]. However, overall, literature support for routine ultrasound use in suspected Boerhaave’s syndrome is very limited [73].

**Figure 3:**
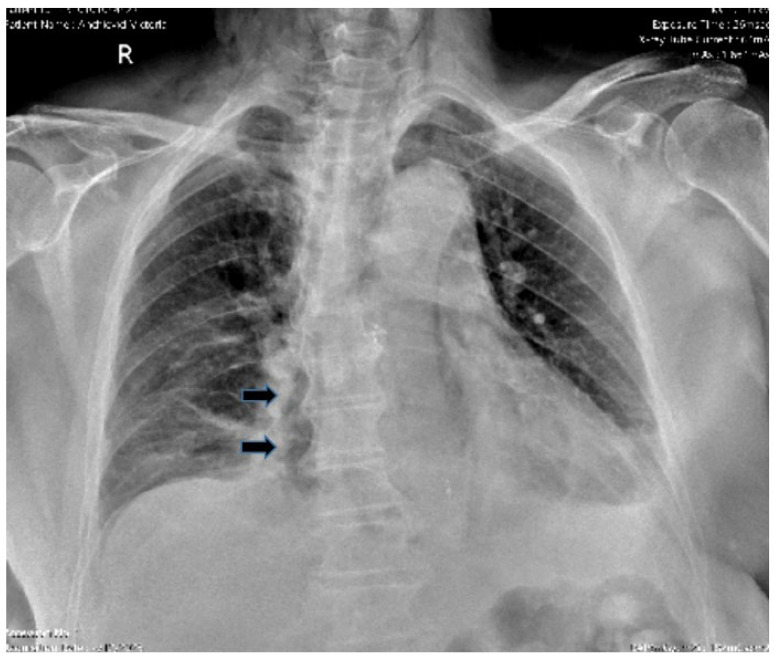
Chest X-ray. Mediastinal aerial image (arrows) is observed that “capes” the esophageal contour, suggestive of an esophageal perforation.

*Chest X-ray* is an accessible and routinely used investigation in emergency medicine and can highlight left-sided pleural effusion, pulmonary infiltrates, atelectasis, pneumomediastinum (Figure 3), or hydropneumothorax [80,81]. Most of these findings are, however, non-specific. Detection of pneumomediastinum is considered suggestive, and in Boerhaave’s syndrome, it may appear radiographically as retrocardiac air bands shaped like a V (Naclerio’s sign), present in about 20% of cases [82,83]. The low sensitivity of chest X-ray in detecting secondary pneumomediastinum is explained by the complex morphological changes caused by digestive leakage into the mediastinum, potentially concealing otherwise specific radiological signs. Moreover, the extent of radiological findings is closely related to the time elapsed since esophageal rupture, volume of mediastinal leakage, and size of the esophageal perforation. Chest X-ray is also essential in the differential diagnosis with perforated gastric or duodenal ulcers by accurately detecting pneumoperitoneum [84].

**Figure 4:**
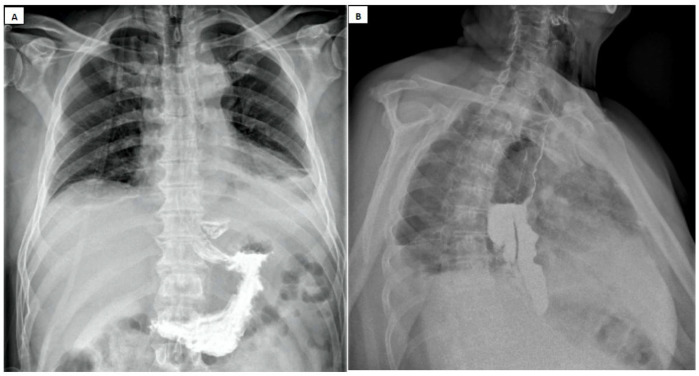
Contrast Swallow Study (water-soluble contrast esophagram). The contrast agent leakage is noted through (**A**) an esophageal rupture of about 1 cm, and (**B**) a large esophageal rupture, about 2–3 cm wide by about 7–8 cm long at the level of the middle third.

*Contrast Swallow Study* is recommended by the American College of Radiology when there is a suspicion of esophageal perforation [85,86]. This investigation (Figure 4) is effective in accurately determining the topography of the lesion and the fistula output [69]. However, 10–20% of esophageal perforations may go undetected on contrast studies when barium is used, and 22–50% when water-soluble contrast agents are used [87,88].

Additionally, contrast esophagography has several limitations when performed in an emergency setting:

- It depends on the availability of qualified, experienced personnel, who may not always be accessible overnight;
- Patient transport to the radiology department, patient cooperation (related to their level of consciousness), correct positioning during the procedure, and swallowing ability are all factors influencing the accuracy of the examination and can be compromised in critically ill patients;
- Barium swallow studies carry a risk of aspiration and pulmonary edema, and residual contrast material in the esophagus may compromise the quality of subsequent upper digestive endoscopy [89].

It is important to note that in cases of suspected esophageal rupture, a water-soluble contrast agent should be used, even though its sensitivity is lower than that of barium, in order to avoid the severe consequences of barium extravasation outside the esophageal lumen. If the initial water-soluble contrast study fails to demonstrate an esophageal perforation, a barium study may be considered subsequently to increase diagnostic accuracy.

*Contrast-enhanced computed tomography (CT)* with oral contrast is the preferred investigation, with a reported sensitivity of 92–100% [69]. It is considered the gold standard for diagnosing spontaneous esophageal rupture [90]. CT provides essential morphological and topographic data about the perforation, and identifies mediastinal collections and signs of mediastinitis [71].

Imaging signs that may be detected on CT include:

- Pneumomediastinum
- Pneumothorax (especially left-sided)
- Pleural effusion (especially left-sided)
- Localized periaortic gas
- Mediastinal fluid collections
- Thickened esophageal wall
- Gas within thoracic soft tissues, neck, or around major vessels
- Gas in the epidural space
- Pneumoperitoneum
- Gas in the retroperitoneal space
- Oral contrast extravasation from the esophageal lumen [10].

However, it is crucial to interpret these imaging findings within the clinical context, as they can also appear in other conditions like spontaneous pneumomediastinum secondary to heavy smoking, drug inhalation, severe coughing, asthma attacks, violent sneezing, COPD, bronchiectasis, lung cancer, foreign body aspiration, childbirth, iatrogenic causes (endoscopy, intubation), or trauma [91].

**Figure 5:**
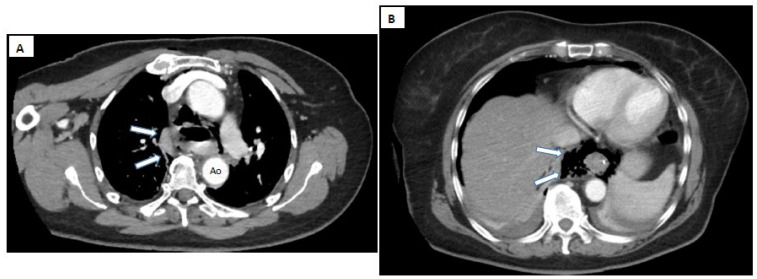
CT scan of the chest (**A**) and upper abdomen (**B**) with contrast agent and cross-sections. Images of a significant hydro-air collection at the mediastinal level (arrows), with extension into the upper abdomen through the esophageal hiatus through an esophageal rupture. Ao—aorta.

**Figure 6:**
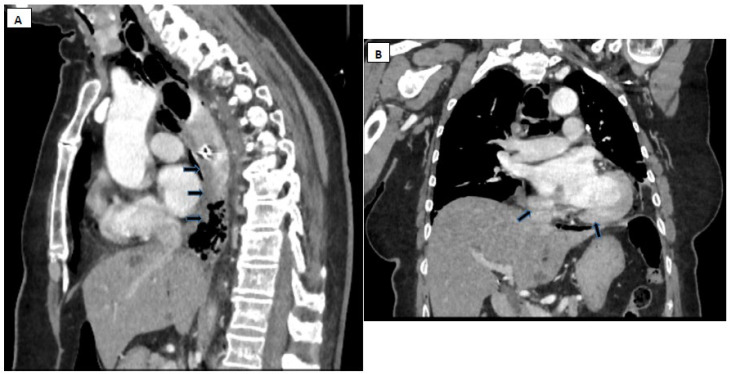
Thoraco-abdominal CT scan with contrast agent, sagittal (**A**) and frontal (**B**) sections. Images of significant mediastinal, supradiaphragmatic fluid collection (arrows) through an esophageal tear in the middle and lower thirds.

CT findings help guide decisions between conservative management or surgical intervention. The CT has the ability to provide rapid and detailed information, without depending heavily on patient cooperation, and its decisive support in navigating complex differential diagnoses make it indispensable in suspected Boerhaave’s syndrome (Figure 5 and Figure 6).

Moreover, CT can sometimes offer information detailed enough to exclude the need for a contrast swallow study (esophagography), saving valuable time for therapeutic management [89]. Nevertheless, a false-positive rate of about 26.3% has been reported for diagnosing pneumomediastinum via CT. In these cases, only radiologic interpretations suggested pneumomediastinum without actual esophageal rupture, confirmed by additional investigations. Contrast swallow studies were negative in all these situations. Therefore, it is suggested that in cases where CT shows isolated pneumomediastinum without other associated findings, further esophagography may be unnecessary [87].

Certain CT characteristics can suggest spontaneous pneumomediastinum rather than secondary to esophageal rupture, such as:

- Air confined to the anterior mediastinum
- Presence of pulmonary emphysema
- Absence of pleural effusion
- Absence of pneumoperitoneum [89].

Thus, identifying these features can help select patients who do not require additional esophageal imaging. Importantly, the large number of false positives should not discourage thorough investigation of any case with radiologic evidence of pneumomediastinum. The real danger lies not in false positives but in false negatives, which can catastrophically delay diagnosis and treatment, worsening the prognosis.

Regarding the esophageal wall lesion, CT is less reliable than contrast swallow studies, with a higher rate of false positives. An important radiologic sign leading to interpretative errors is the presence of intramural esophageal air, mistakenly interpreted as perforation in about 15.7% of cases. It is speculated that these findings may reflect Mallory-Weiss tears rather than full-thickness perforations, with the lack of confirmation possibly due to the absence of endoscopic studies [89]. Further studies correlating CT findings with endoscopic data are needed to clarify the significance of intra-parietal gas images.

Additionally, beyond mediastinal assessment, CT is useful for detecting subcutaneous cervical emphysema with up to 64% accuracy, an important clue in diagnosing Boerhaave’s syndrome [84].

**Figure 7:**
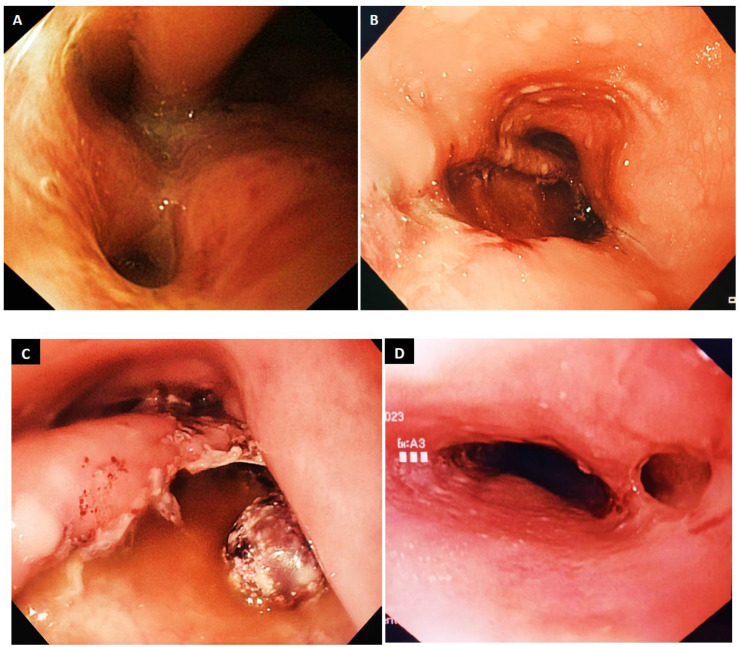
Upper endoscopy images. Different types of lesions due to Boerhaave-type esophageal rupture, with varying sizes and parietal involvement. (**A**) Esophageal rupture of about 5–6 mm, in the distal esophagus, approximately 5 days after development. (**B**) Extended esophageal lesion, approximately 2–3 cm wide by approximately 7–8 cm long at the level of the middle third, after 24 h of development. (**C**) Rupture in the lower esophagus, with necrosis and remnants of esophageal wall, 4 days after the etiological event. (**D**) Small rupture, 3–4 mm, after an episode of severe vomiting, on the pathological esophagus (eosinophilic esophagitis).

*Upper GI Endoscopy* has high sensitivity and specificity for diagnosing esophageal lesions. However, it must be acknowledged from the outset that it can worsen the esophageal injury and increase mediastinal contamination if a perforation is present [92] (Figure 7).

### Alternative Diagnosis Clues

Practically, the presence of mediastinitis associated with a recent history of vomiting followed by chest pain is considered practically pathognomonic for Boerhaave’s syndrome [93]. In some cases, diagnosis can be made unequivocally.

Examples include:

- Methylene blue test—When oral methylene blue administered to the patient appears through a pleural drainage tube, the diagnosis of esophageal perforation is confirmed.
- Thoracocentesis findings—Suzuki et al. report a case of Boerhaave’s syndrome diagnosed by thoracocentesis, revealing bloody fluid and food debris, confirming the diagnosis beyond doubt [94].
- Other thoracocentesis clues—A high level of amylase and pleural fluid pH < 6 are also considered suggestive for esophageal perforation [71].

## 9. Treatment

The therapeutic management of Boerhaave’s syndrome consists of two main pillars:

(1) Supportive care, usually provided in the Intensive Care Unit (ICU), which includes vital function support, broad-spectrum antibiotic therapy, proton pump inhibitors (PPI), parenteral nutrition;
(2) Definitive management of the esophageal wall breach, aimed at closing the esophageal perforation.

These two treatment directions must be approached simultaneously, and are directly influenced by factors such as the morphology of the perforation, the extent of contamination, the time elapsed since perforation, and the presence of preexisting comorbidities.

### 9.1. Conservative Treatment

Minimal contamination may qualify a patient for conservative management, which consists of: nil per os (oral intake restriction), antibiotic therapy, parenteral nutrition, close monitoring, drainage of fluid collections if they form. This approach is reserved for carefully selected cases. However, even under these conditions, it is important to note that 51.2% of patients undergoing conservative management may eventually require surgical intervention [95].

### 9.2. Antimicrobial and Antifungal Therapy

Septic mediastinitis is often caused by polymicrobial infections, including aerobic and anaerobic bacteria. Broad-spectrum antibiotics are essential in the initial management to cover potential pathogens, including methicillin-resistant *Staphylococcus aureus* (MRSA) and Gram-negative bacteria [49]. Choice of antimicrobials should be guided by local resistance patterns and subsequently de-escalated based on culture results. Early empiric antibiotic therapy represents a critical pillar in the management of septic mediastinitis in Boerhaave’s syndrome. The antibiotic regimen should cover both aerobic and anaerobic organisms. A typical combination includes a broad-spectrum beta-lactam (e.g., piperacillin/tazobactam or meropenem) and an anti-MRSA agent (e.g., vancomycin or an oxazolidinone). In cases of penicillin allergy, clindamycin in combination with fluoroquinolones and aminoglycosides can be considered. Fungal infections, especially candidiasis, are common in critically ill patients with septic mediastinitis. Antifungal therapy should be considered in patients with refractory shock or risk factors for fungal infections (such as immunosuppression, diabetes, obesity, or chronic renal failure) [96]. Fluconazole or echinocandins are recommended as first-line antifungal agents, depending on the suspected pathogen and local resistance patterns.

The duration of antibiotic therapy in septic mediastinitis is typically 10–14 days, depending on the clinical response and the presence of complications like abscesses or empyema. Extended courses of up to 2–4 weeks may be required in patients with persistent infection [49]. Patients with significant contamination require endoscopic and/or surgical intervention.

Endoscopic treatment (endoluminal therapy) in spontaneous esophageal perforation is complex, with the objectives of ensuring esophageal patency, performing antiseptic lavage at the site, and closing the wall defect. The treatment methods include: placement of fully covered esophageal stents, through-the-scope clips (TTS), over-the-scope clips (OTSC), endoscopic suturing, and vacuum therapy [97].

Radiologic control with iodinated contrast agent, both peri- and post-procedurally, is essential for monitoring the efficacy of the endoscopic approach and documenting the closure of the esophageal perforation [98]. The choice of endoscopic technique depends on the size, location, and extent of the defect, as well as the quality of the defect’s margins. Moreover, the clinical impact of mediastinitis, the general condition of the patient, pre-existing comorbidities, and the time elapsed before treatment initiation must not be overlooked [99]. Significantly, interventional endoscopic maneuvers require advanced expertise and are generally limited to cases without extensive lesions or severe mediastinitis.

### 9.3. Clip Placement

TTS clips are recommended for patients showing endoscopic signs of transmural esophageal laceration, provided the general condition is good and thoraco-abdominal CT imaging with contrast does not reveal collections or periesophageal necrosis [100]. TTS clips are indicated for spontaneous esophageal perforations up to 10 mm in diameter, while OTSCs can be used for perforations up to 20 mm [101]. OTSCs are preferred over fully covered esophageal stents for perforations smaller than 10 mm, particularly when there is gastric extension or rapid diagnosis of perforation [102]. A multicenter retrospective study involving 188 patients with gastrointestinal wall defects, including 10 esophageal perforations, demonstrated a 100% success rate for OTSC in esophageal perforations [9]. In patients with poor general condition or severe comorbidities, when the perforation diameter does not exceed 20 mm, an OTSC followed by the placement of a fully covered esophageal stent may be utilized [103].

### 9.4. Stent Placement

Fully covered esophageal stents represent a safe and highly effective method for treating esophageal perforations, particularly when the diameter exceeds 20 mm [104]. It is recommended that fully covered stents be maintained for 4–6 weeks, with mandatory removal at no later than 6 weeks, especially in large perforations [105]. Ineffective stenting is indicated by the persistence of an esophageal fistula on post-procedural iodine-based contrast imaging, which may necessitate placement of a larger diameter stent (restenting) or repositioning in cases of migration [106]. Perforations located at the upper or lower esophageal sphincter or transmural defects larger than 60 mm are associated with poor outcomes for esophageal stenting [107].

The technical success rate of esophageal stenting reaches up to 91.4% [108]. The efficacy of fully covered stents as adjuncts to surgery is well-documented, and they may also serve as a standalone treatment in carefully selected patients, although this approach remains highly controversial [109,110,111]. Dasari et al. [108] emphasize that endoscopic esophageal stenting alone does not fully address the therapeutic protocol’s tactical goals.

This view is supported by other studies showing that 82% of patients receiving a stent eventually required additional interventions, such as feeding jejunostomy, to adequately manage the case [112]. The complications associated with fully covered esophageal stent placement include: stent migration (25% rate), retrosternal pain, gastrointestinal bleeding at the proximal or distal ends of the stent, esophageal perforation, aorto-esophageal fistula, tracheo-esophageal fistula. Such a case with tracheo-esophageal fistula has been described in a patient treated for BS [7,113]. Although rare, occurring in about 4% of cases, the incidence increases proportionally with the duration of stent placement.

*Endoscopic Therapy with Vacuum* involves the placement of a spongy material intraluminally at the level of the transmural defect, followed by negative pressure suction through a tube positioned within the sponge material. Applying negative pressure to the cavity promotes closure of the esophageal wall defect through the formation of granulation tissue [114]. This technique was developed in esophageal surgery for controlling esophageal fistulas, but there are few reports of its use in cases of BS. In the absence of closure of esophageal perforations after endoscopic stent removal, endoscopic vacuum therapy can stimulate granulation and lead to closure of the esophageal defect. A specific disadvantage of endoscopic vacuum therapy is the need to replace the sponge material every 3–4 days [115].

### 9.5. Combined Vacuum-Stent Therapy

The Vacuum-Stent is a new endoscopic technique that combines the advantages of endoscopic vacuum therapy with a fully covered intraluminal esophageal stent. It is indicated for large esophageal defects, with or without associated collections [116]. It is a therapeutic option that depends on the morphological characteristics of the esophageal wound and the extent of mediastinal contamination [117]. In combination with esophageal stenting, the placement of a vacuum system can prevent stent migration and facilitate healing through the application of negative pressure [118]. Most often, the vacuum system is used when there is failure after an initial suturing attempt and recurrence of the esophageal fistula [119]. This new endoscopic technique allows closure of the esophageal perforation/defect through continuous suction that stimulates granulation tissue formation, while allowing enteral feeding [120].

The disadvantages of Vacuum-Stent therapy include: (1) a technically demanding procedure that requires specialized training for both the endoscopist and the assistant, (2) the need to replace the Vac-Stent system every 5–7 days, (3) quality of life discomfort due to the presence of a nasal tube, (4) relatively high cost [121].

*Endoscopic suturing* is a newly developed endoscopic technique, mainly indicated for closing mucosal defects after endoscopic submucosal dissection (ESD) and for iatrogenic perforations of the digestive tract occurring during endoscopic procedures. To close spontaneous esophageal perforations, sutures must be placed on the healthy margins of the esophageal mucosa [122]. The temporary limitations of this method are the technical difficulty and the high cost of the procedure [123].

## 10. Surgical Management

*Primary closure.* In 1947, Barrett reported the first successfully surgically treated case of BS. The technique used was primary suture of the esophageal rupture with catgut [124]. In most cases, surgical access is achieved via a right posterolateral thoracotomy, which provides good exposure—especially to the proximal and middle esophagus. For lesions of the distal esophagus, a left thoracotomy may be preferred. A wide incision of the mediastinal pleura allows for debridement of devitalized tissue and adequate drainage of the mediastinum, a key component in controlling mediastinitis. Minimally invasive techniques, such as thoracoscopy or video-assisted thoracotomy, have been described in selected centers, primarily in hemodynamically stable patients, provided that the exposure is sufficient to allow accurate visualization and repair of the esophageal lesion [25].

Primary suture is generally considered the standard technique in such lesions. Reinforcement techniques aim to reduce the incidence of fistulas. Primary suture with a patch of healthy adjacent tissue is considered the most reliable approach, associated with the lowest morbidity and mortality rates [69]. Various reinforcement materials may be used, including: (i) Pleural or pericardial flaps, (ii) Epiploic patch, (iii) Pedicled extrathoracic muscle flaps, (iv) Pedicled diaphragmatic flap, (v) Gastric fundoplication [17,95,125].

Pleural or intercostal muscle patches are most commonly used for thoracic esophageal lesions. The pleural flap is a well-established and technically simple method—in a right thoracic approach, the pleura is incised in a U-shape around the perforation and dissected anteriorly from the azygos vein and right intercostal veins. In left thoracotomy, the flap is dissected anteriorly from the aorta and left intercostal vessels. Some studies report that muscle patches may reduce fistula rates in patients with perforations older than 24 h who undergo delayed primary suture [126,127]. However, to improve outcomes, the muscle flap must be macroscopically healthy, without hyperemia, edema, or contamination [128].

Epiploic or gastric wall patches are typically used for abdominal or lower thoracic esophageal lesions. The epiploic flap is mobilized through a radial phrenotomy and coloepiploic dissection for left thoracotomies. In right thoracotomies, a retrosternal tunnel is created to advance the flap into the thoracic cavity. The gastric flap is specific for lower thoracic esophageal perforations. It is accessed through left thoracotomy, requires phrenotomy, and has notable advantages: high resistance, good mobility, and rich vascularization [129].

Not all studies confirm a lower fistula rate when using reinforcement techniques with adjacent structures. This outcome is directly correlated with the interval between perforation and surgical treatment. Importantly, the 8-hour threshold from symptom onset to surgical suture is a critical window. Exceeding this window leads to an exponential increase in the risk of esophageal fistula after primary repair. The incidence of esophageal fistula following primary suture is estimated at approximately 40% [17]. If primary suture is not feasible due to extensive tissue damage, alternative surgical approaches must be considered [69].

*T-Tube Insertion* (Controlled Esophago-Cutaneous Fistula).

When tissue necrosis with massive contamination occurs in late-presenting cases, T-tube drainage can be a solution. The goal is to create a controlled esophago-cutaneous fistula to drain the esophagus and allow healing of surrounding tissues. A survival rate of approximately 70% is estimated with this technique [130,131].

**Figure 8:**
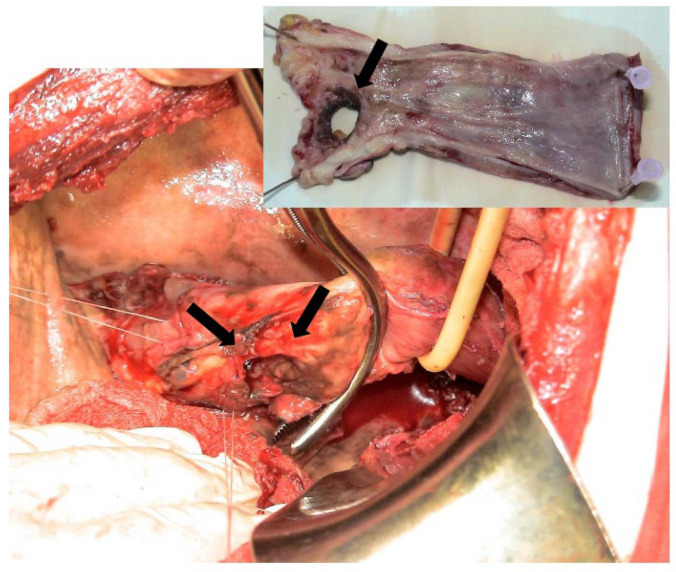
Intraoperative image—open surgery. A large Boerhaave-type esophageal perforation (arrows) is observed, with necrotic margins, with significant mediastinal reaction (remnants of esophageal wall, false membranes), with severe sepsis. The smaller image shows the subtotal esophagectomy specimen.

*Esophagectomy.* Esophagectomy is generally considered a last-resort solution in BS due to the magnitude of the surgical intervention. In some cases, the extent of the esophageal rupture clearly renders other techniques ineffective from the outset. The decision is complex and depends on numerous factors: the time elapsed since diagnosis, patient comorbidities, the optimal surgical approach (open vs. minimally invasive), and the experience of each surgical team (Figure 8). Ultimately, the intraoperative findings guide the final decision. The surgeon must assess the lesion (size, mediastinal involvement, time since perforation, etc.) and determine the most appropriate technical option: primary suture (with or without reinforcement), type and placement of drainage, whether esophagectomy is indicated, and whether it should be performed in one stage or two [127,132,133,134]. The procedure may be open or minimally invasive. In two-stage surgery, digestive tract reconstruction is done later, based on patient recovery. For example, in one case [135], gastroplasty was performed just 3 days after esophagectomy, a very “bold” approach.

### Esophageal Diversion (Exclusion)

Esophageal exclusion is an exceptional therapeutic option, considered when primary closure of the rupture is not feasible due to the extent of the lesion or delay in diagnosis. The approach involves excluding the esophagus from digestive transit via a cervical esophagostomy, placement of a feeding route (gastrostomy or jejunostomy), and a delayed reconstruction. Exclusion begins with lower esophageal transection (via thoracic or abdominal approach), followed by cervical esophageal exclusion through a left cervicotomy. It is critical to mobilize the cervical esophagus as far away from the mediastinum as possible [136]. Simultaneously, control of mediastinitis is required via pleural and mediastinal drainage and debridement of necrotic tissues.

For an overview of the issue, in a large Japanese study (95 treated cases), primary suture was performed in 78.9% of patients, T-tube drainage in 15.85% of cases, and esophagectomy in 5.3%. The low mortality rate of only 3.64% was mainly attributed to the prompt management of the cases and the optimal selection of the treatment approach [95].

*The Mixed Approach* (Hybrid Techniques). The use of esophageal stenting has gained significant ground in the treatment of esophageal perforations. Unfortunately, the presence of mediastinal collections and the inability to manage them solely through endotherapy approach often necessitate the addition of a surgical technique, leading to a so-called hybrid management strategy. The advantages are clear: on one hand, stenting or other endoscopic modalities prevent further mediastinal contamination and ensure esophageal sealing; on the other hand, surgery addresses the consequences of mediastinal or pleural contamination—most often through minimally invasive techniques [137].

**Figure 9:**
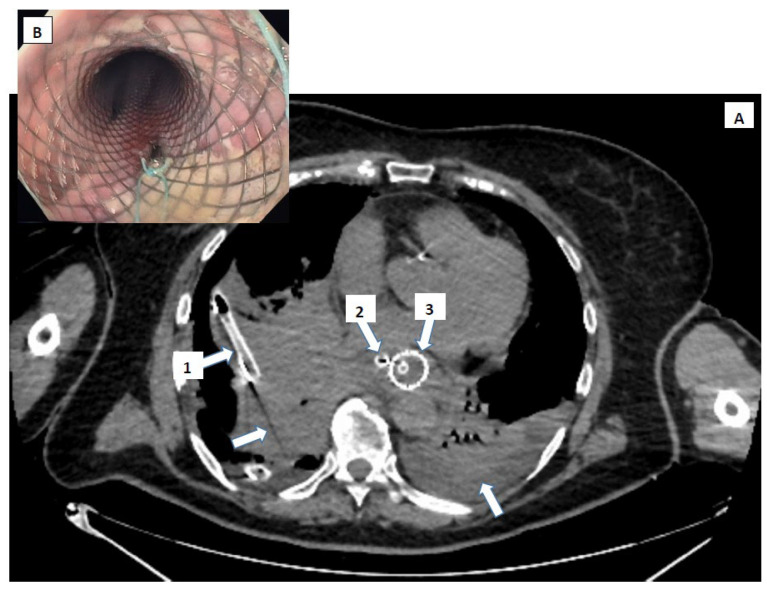
Post-therapeutic control tomography—cross-section (**A**). Thoraco-laparoscopic and interventional gastro-enterological surgical multidisciplinary approach, with the evidence of a large hydro-air collection at the mediastinal level and in the right pleural cavity. Note (1) the drain tube (arrow 1) from the pleural collection, (2) the mediastinal drain tube (arrow 2) exteriorized transhiatally and abdominally, and (3) the esophagus with a fully covered stent (arrow 3) with a nasogastric suction probe passed. The smaller image (**B**) shows the post-procedural stent.

This unified, “rendez-vous” approach is performed during a single operative session under selective intubation anesthesia. The endoscopist easily identifies the esophageal lesion, thereby facilitating the surgeon’s direct access to the site of the esophageal rupture during the thoracic approach—a direct transpleural approach to the esophageal tear and the mediastinal space. Thorough lavage, wide debridement, and consistent irrigation followed by esophageal suturing are essential steps. In cases where the local impact of the lesion is moderate, a simpler strategy involving thoracic cavity drainage may be sufficient. This can be complemented by a laparoscopic transhiatal approach to the mediastinum, allowing for additional debridement, lavage, and external drainage. The final step will be taken by the endoscopist, who will complete the procedure by sealing the esophagus through stenting for another endoluminal option [97] (Figure 9). The results appear encouraging, making this one of the viable management options for BS.

## 11. Discussions

BS is a pathological entity that presents numerous challenges. Diagnosis is not always easy and not only diagnostic errors but even the delay in establishing it has dramatic consequences on the prognosis. Most importantly, once the diagnosis is identified, the description and cataloging of the lesional anatomy appears, being the guide to the therapeutic solution. Subsequently, the mediastinal and/or thoracopulmonary impact as well as the systemic one—the presence or absence of sepsis—will also intervene in the therapeutic decision.

Therapeutic management is based on two important objectives: repair of the esophageal rupture and management of secondary mediastinitis. Obviously, the two etiopathogenic elements are directly related from the beginning, but from a certain point in evolution, the mediastinitis detaches from the initial cause, evolving towards worsening independent of resolving the esophageal integrity and interrupting the esophageal contamination.

Although the literature clearly shows that endoscopic techniques are the first to be considered, most of the time an approach is necessary that is permanently adapted to the patient’s evolution, and also combines in different forms the available techniques (endoscopic and surgical, classical or minimally invasive).

In this direction, Schweigert et al. evaluates a group of 13 patients with BS who were treated by initial stenting. In 85% of them, additional surgical techniques were subsequently required due to the development of complications such as pleural empyema or mediastinal abscesses [138]. Biancari et al. confirm the high rate of additional interventions after stenting, such as pleural drainage or thoracoscopy in 83% of cases [139]. Moreover, they report the need for endoscopic reinterventions for stent migration in 59% of cases or fistula persistence in 33% of cases.

In fact, unlike benign esophageal strictures or esophageal cancer, stent migration occurs more frequently in patients with benign esophageal perforations, as a result of insufficient support from the esophageal wall. In this regard, numerous techniques have been proposed to anchor the stent and minimize the risk of migration, including the use of clips or suture fixation [140]. To minimize the risk of migration and insufficient sealing, appropriate stent management appears essential, namely the careful study of the lesional anatomy that will allow for the most appropriate selection (fit for lesion) in terms of dimensions (thickness, length) and type of stent used.

A 10-year follow-up of patients treated for BS has shown lower mortality and shorter hospital stays for those treated endoscopically compared with those treated surgically. On the other hand, patients treated exclusively endoscopically had a higher number of readmissions than those who underwent surgery [141].

Closure of the esophageal wound by applying clips is reserved for patients with early diagnosis, stable, without signs of infection or mediastinal contamination. Although an initial success rate of up to 76% is reported, subsequent failure is described in 75% of cases, with clip failure requiring surgical intervention. Better results are obtained for ruptures of up to 20 mm. Often, a combined technique of clipping and simultaneous installation of a metallic stent is adopted [142,143].

In principle, stents are recommended for esophageal ruptures up to 70% of the circumference, while larger lesions are addressed to surgery. It should be noted that the stenting technique is also used in cases of surgical failure, in patients who do not qualify for re-surgical intervention [143,144].

The combination of stenting and thoracoscopy for drainage and mediastinal debridement is a tactic promoted by some teams, which report favorable results in selected cases. Although even with such an approach the rate of surgical re-interventions remains at approximately 42%, these can still be effectively performed through thoracoscopy. It should be noted that even in these situations, stent migration or fistula persistence remains a problem, reported in 67% of cases [140].

The combined approach of endoscopic stenting and thoracoscopic debridement may be limited in the case of cervical or very distal perforations near the esophagogastric junction, as endoscopic stent placement may be technically difficult in these locations. Furthermore, the Boerhaave lesion is not specific for high esophageal hiatus hernias. Furthermore, adhesions after previous thoracic surgeries may complicate a minimally invasive thoracic approach. Obviously, thoracotomy may be an option in these situations. A particular aspect is ensuring an adequate nutritional intake in these patients until oral intake is resumed. Total parenteral nutrition has many disadvantages, especially from the perspective of these patients in whom oral intake may not be available for a long period of time.

**Figure 10:**
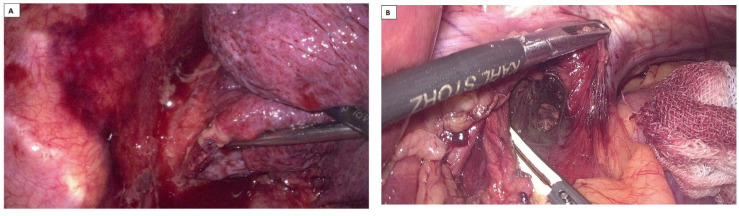
Boerhaave-type esophageal perforation; intraoperative images of minimally invasive surgery in a hybrid treatment. (**A**) First stage—thoracoscopic approach to hematic pleural collection through mediastinal pleural effusion, with pus, debris and tissue necrosis. (**B**) Second stage—transhiatal laparoscopic approach to the mediastinal collection.

The placement of a feeding jejunostomy allows immediate enteral nutrition, and some authors have also reported the additional use of a decompression gastrostomy to control the fistula flow at the mediastinal level in the treatment of iatrogenic or spontaneous esophageal perforations in selected patients [25]. From this perspective, a combined, simultaneous approach of endoscopic stenting and minimally invasive surgery offers the possibility of sealing the esophageal rupture, concomitantly with thoracoscopic drainage and laparoscopic transhiatal mediastinal drainage (Figure 10). The minimally invasive abdominal approach presents a reasonable aggression compared to the benefit of mediastinal drainage, additionally offering the possibility of installing a feeding jejunostomy at the end of the operation.

**Figure 11:**
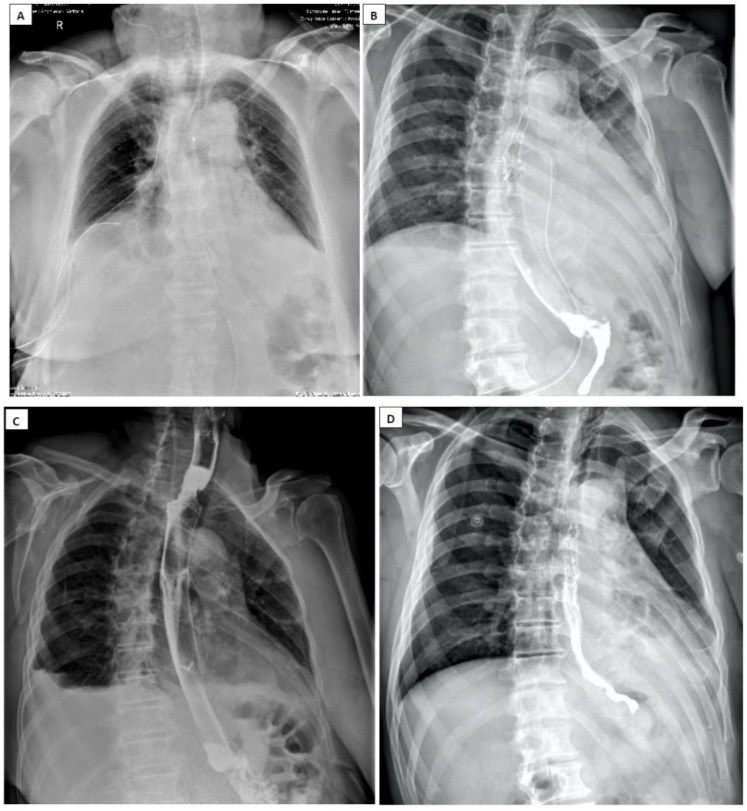
Post-therapeutic periodic evaluation radiology. (**A**) Standard radiology 24 h after multidisciplinary intervention, thoraco-laparoscopic surgery and interventional gastroenterology. The stent is noted in the correct position, pleural and mediastinal drainage. (**B**) Contrast radiology at 5 days, stent in place, no images of esophageal leakage in the mediastinum. (**C**) Contrast radiology at 14 days, stent well placed, in place, no drainage. (**D**) Contrast radiology after stent extraction, with normal progression and passage of contrast material, no esophageal leakage.

No significant differences in efficacy have been described between metallic and plastic stents [145]. Long-term studies have documented a higher risk of stent migration in patients with BS, ranging from 10 to 37%, and their fixation is therefore recommended [146,147]. Postprocedural patient monitoring is also done from two perspectives: esophageal wall restraint to ensure the absence of extravasation of digestive contents from the esophageal lumen to the mediastinum, and the healing of the initial mediastinitis foci (Figure 11).

Contrast-enhanced radiological transit and oral contrast-enhanced chest CT are essential investigations that need to be performed in dynamics, according to a protocol adapted to each case, depending on the evolution. It should not be forgotten that false-negative results for contrast-enhanced radiological examination have a high incidence, up to 10–38%. Therefore, correlation with clinical and biological data is essential. It is documented that a CT scan of the chest with oral contrast medium provides useful information regarding both esophageal rupture and periesophageal changes [148].

Antibiotic treatment is mandatory; it needs to cover aerobic, anaerobic and fungal flora, for a duration that varies depending on the evolution of the case from a minimum of 10–14 days, up to 4 weeks for severe cases [49].

Stent removal is performed after 5–6 weeks. Exceeding this period makes extraction difficult and risky due to scar tissue that encompasses the device [146].

The prognosis of BS depends largely on the duration of symptoms and the early recognition and management of the esophageal lesion. The Pittsburgh Severity Score summarizes these parameters, quantifies the severity of the clinical presentation for each patient, and can guide an individualized therapeutic pathway [149]. The score was developed in 2009 by Abbas et al. to estimate the clinical severity of esophageal perforation [150]. Age, heart rate, leukocytosis, presence of pleural effusion, fever, hypotension, uncontrolled esophageal fistula, time since diagnosis, respiratory distress, and associated neoplasia diagnosis are all important prognostic factors, taken into account in this score, evaluated with 1–3 points, with a maximum score of 18 points.

The Pittsburgh score has been shown to be significantly predictive of complications after spontaneous esophageal perforation and its use has been supported in the literature as a tool in the initial evaluation of these patients [149]. Schweigert et al. [66] documented that mortality is correlated with the Pittsburgh Severity Score (PSS), with a mortality rate of 3.2% for a PSS of 2 or less, 7% for a PSS of 3 to 5, and 37.5% for a PSS of 5 or more [69].

The Comprehensive Complication Index (CCI) [151], which is a scale from 0 (no complications) to 100 (death), is higher in patients who have undergone esophagectomy than in those who have undergone primary suturing. It is unclear, however, whether this result is due to comorbidities, surgical technique, or other factors.

## 12. Conclusions

Boerhaave syndrome and subsequent septic mediastinitis represent a complex cascade of events from esophageal perforation to septic shock. The pathophysiology involves chemical injury, polymicrobial contamination, cytokine storm, endothelial dysfunction, coagulation disorders, and ultimately multiple organ failure. Understanding these mechanisms is crucial for the development of targeted interventions that can interrupt this lethal sequence.

The time frame for successful treatment is narrow, with outcomes directly related to the time to diagnosis and intervention. Primary surgical repair of esophageal perforation within the first 24 h of symptom onset offers the best prognosis, with mortality rates increasing dramatically beyond this interval.

Due to the complexity, it is almost impossible to develop a feasible treatment protocol in every situation. Understanding the pathophysiological mechanisms and consequences of spontaneous esophageal rupture is the basis for the management of these cases, which involves adapting and customizing the treatment for each individual situation. The available techniques (endoscopic, surgical, classical or minimally invasive) for resolving esophageal rupture assume, on one hand, the availability of sophisticated, expensive equipment, and on the other hand, that they are addressed to multidisciplinary teams (gastroenterologist, surgeon) with experience in esophageal pathology. Moreover, mediastinitis secondary to extraluminal digestive spillage, in addition to its formal severity, can evolve independently even after restoring the integrity of the esophageal wall, so that the involvement of an Intensive Care team with experience in esophageal pathology is essential.

## Data Availability

No new data were created or analyzed in this study. Data sharing is not applicable to this article.
